# Genome-wide identification of the C2H2 zinc finger gene family and expression analysis under salt stress in sweetpotato

**DOI:** 10.3389/fpls.2023.1301848

**Published:** 2023-12-13

**Authors:** Taifeng Du, Yuanyuan Zhou, Zhen Qin, Aixian Li, Qingmei Wang, Zongyun Li, Fuyun Hou, Liming Zhang

**Affiliations:** ^1^ Key Laboratory of Phylogeny and Comparative Genomics of the Jiangsu Province, School of Life Sciences, Jiangsu Normal University, Xuzhou, China; ^2^ Crop Research Institute, Shandong Academy of Agricultural Sciences/Scientific Observing and Experimental Station of Tuber and Root Crops in Huang-Huai-Hai Region, Ministry of Agriculture and Rural Affairs, Jinan, China

**Keywords:** salt stress, C2H2-Zinc finger protein, expression profiles, synteny analysis, sweetpotato

## Abstract

**Introduction:**

The higher plant transcription factor C2H2 zinc finger protein (C2H2-ZFP) is essential for plant growth, development, and stress response. There are limited studies on *C2H2-ZFP* genes in sweetpotato, despite a substantial number of *C2H2-ZFP* genes having been systematically found in plants.

**Methods:**

In this work, 178 *C2H2-ZFP* genes were found in sweetpotato, distributed randomly on 15 chromosomes, and given new names according to where they were located. These members of the zinc finger gene family are separated into six branches, as shown by the phylogenetic tree. 24 tandem repeats of *IbZFP* genes and 46 fragment repeats were identified, and a homology study revealed that *IbZFP* genes linked more regions with wild relative species of sweetpotato as well as rhizome plants like potato and cassava. And we analyzed the expression patterns of *IbZFP* genes during the early development of sweetpotato storage roots (SRs) and salt stress using transcriptome data, and identified 44 *IbZFP* genes that exhibited differences in expression levels during the early expansion of sweetpotato SRs in different varieties, and 92 *IbZFP* genes that exhibited differences in expression levels under salt stress in salt tolerant and salt sensitive sweetpotato varieties. Additionally, we cloned six *IbZFP* genes in sweetpotato and analyzed their expression patterns in different tissues, their expression patterns under abiotic stress and hormone treatment, and subcellular localization.

**Results and discussion:**

The results showed that the *IbZFP* genes had tissue specificity in sweetpotato and were induced to varying degrees by drought and salt stress. ABA and GA_3_ treatments also affected the expression of the *IbZFP* genes. We selected *IbZFP105*, which showed significant differences in expression levels under salt stress and ABA treatment, to be heterologously expressed in *Arabidopsis thaliana*. We found that *IbZFP105* OE lines exhibited higher tolerance to salt stress and ABA stress. This indicates that *IbZFP105* can enhance the salt tolerance of plants. These results systematically identified the evolution and expression patterns of members of the *C2H2-ZFP* gene family in sweetpotato, providing a theoretical basis for studying the role of *IbZFP* genes in the development of sweetpotato SRs and in resistance to stress.

## 1 Introduction

Sweetpotato (*Ipomoea batatas* (L.) Lam.) is an important food crop with ultra-high yield characteristics, and sweetpotato is rich in protein, dietary fiber, polyphenols, vitamins, mineral elements, and other nutrients needed by the human body. It is one of the globally recognized nutrients (Neela and Fanta, 2019). The sweetpotato planting area can reach 7.4 million hectares, with an annual production of about 88.9 million tonnes of storage roots (SRs). As the main edible tissue of sweetpotato, the production and development of SR are their most important agronomic traits (Du et al., 2023). However, adverse abiotic factors like salt and drought severely restrict the sweetpotato plant’s growth, development, and output (Zhang et al., 2023). Transcription factors play an important regulatory role in the transmission of plant stress signals, regulating the expression of multiple stress related genes and improving plant stress resistance. The regulatory effects of many transcription factors have been reported, for example, the MYB transcription factor (Dubos et al., 2010; Li et al., 2016), the WRKY transcription factor (Chen et al., 2009; Raineri et al., 2015), the NAC transcription factor (Yuan et al., 2019; Diao et al., 2020), the bHLH transcription factor (Guo et al., 2021; Qian et al., 2021), the bZIP transcription factor (Joo et al., 2021; Han et al., 2023), and zinc finger transcription factor (Kiełbowicz-Matuk, 2012; Yin et al., 2017), *etc*. Zinc finger proteins (ZFPs) the largest transcription factor family in plants, are present in many different species. In controlling plant growth and development and responding to varied environmental challenges, they serve a critical regulatory role (Han et al., 2021).

ZFPs can be categorized into many subfamilies, such as C2H2, C3H, C3HC4, etc., depending on the quantity and arrangement of cysteine and histidine residues in their secondary structure. C2H2 zinc finger proteins (C2H2-ZFPs) are one of them that have undergone more in-depth study (Jiao et al., 2020). The *EPF1* gene of Petunia is the earliest zinc finger protein found in plants with a C2H2 zinc finger structure (Takatsuji et al., 1992). Numerous C2H2-ZFPs have so far been found in plants, including *Arabidopsis thaliana* 176 (Englbrecht et al., 2004), *Sorghum bicolor* 145 (Cui et al., 2022), *Vitis vinifera* 98 (Arrey-Salas et al., 2021), and *Panax ginseng* 115 (Jiang et al., 2022). The length of the long spacer between the two zinc fingers in plant-specific C2H2-ZFP (Q-type C2H2-ZFP) is different from that of other eukaryotes (Ciftci-Yilmaz and Mittler, 2008). A highly conservative QALGGH sequence is typically found in Q-type C2H2-ZFPs, allowing ZFPs to detect target genes and control their expression levels (Wang et al., 2019). There are several distinct classification types and standards used for C2H2-ZFPs. Usually, the number of zinc finger domains, the spacing between them, the series or dispersion of zinc finger domains, and the QALGGH sequence are used to categorize C2H2-ZFPs (Liu et al., 2022b).

*C2H2-ZFP* genes can regulate plants to cope with various abiotic stresses such as high salinity, drought, cold, etc. and plays a very important role in plant adaptation to the environment (Han et al., 2020). C2H2-ZFPs can typically interact with plant hormones to affect the phenotype of plants under stress. (Kiełbowicz-Matuk, 2012; Liu et al., 2022b). For example, overexpression of *OsZFP179* enhances the salt tolerance of rice, transgenic seedlings show hypersensitivity to exogenous ABA (Sun et al., 2010), *OsZFP36* is a key participant in rice abscisic acid induced antioxidant defense and oxidative stress tolerance (Zhang et al., 2014), and *PtrZPT2-1* encodes C2H2-ZFP from *Poncirus trifoliata*, which can enhance the tolerance of plants to various abiotic stresses (Liu et al., 2017).

In addition, C2H2-ZFP can also participate in the growth and development of many plant organs and structures. For instance, during seed germination and plant development, *Arabidopsis thaliana AtZFP3* interferes with the transmission of abscisic acid and light signals (Joseph et al., 2014), *Arabidopsis thaliana AtZFP1* acts upstream of the key trichome initiation factors GL3 and TRY, and overexpression of *AtZFP1* significantly increases the number of trichomes on the stem, leaf, lateral branch, and main stem (Zhang et al., 2020). *AtZFP5* is associated with ethylene signaling and regulates root hair development induced by phosphate and potassium deficiency in *Arabidopsis thaliana* (Huang et al., 2020).

In this study, we identified 178 C2H2 zinc-finger proteins (IbZFPs) from the genome of sweetpotato and analyzed their phylogenetic relationship, chromosome location, collinearity with other species, gene structure, conservative motif promoter cis-regulatory element, and subcellular location. In addition, we also analyzed the expression profile of *IbZFP* genes in sweetpotato SR development and salt stress. Some *IbZFP* genes were detected by real-time quantitative polymerase chain reaction (qRT-PCR). And overexpression of *IbZFP105* in *Arabidopsis thaliana* was revealed to enhance its tolerance to salt and ABA stress. These results should provide a very important theoretical basis for studying the function of *IbZFP* genes in the future.

## 2 Materials and methods

### 2.1 Identification of C2H2-ZFPs in sweetpotato

The hexaploid sweetpotato Taizhong6 genome sequence was extracted from the Ipomoea Genome Hub database (https://sweetpotao.com/download_genome.html, accessed on 7 March 2023). In order to screen the possible *C2H2-ZFP* coding genes in the genome, two methods were used. The HMM profile of the C2H2-ZFP domain (PF00096, PF13894, PF13912, PF18414, and PF16622) was downloaded from the Pfam database (http://www.ebi.ac.uk/interpro/, accessed on 8 March 2023) (Paysan-Lafosse et al., 2023) and was used to identify the *C2H2-ZFP* genes in the *Ipomoea batatas* genome using HMMER 3.0 software with an E value < 1e^-5^. Using all AtZFPs in the *Arabidopsis thaliana* genome database (https://www.Arabidopsisthaliana.org/index.jsp, accessed on 7 March 2023) as queries, the BLAST algorithm identifies the predicted IbZFPs (BLASTP, E value < 1e^−5^). All the protein sequences obtained by the two methods were subjected to domain analyses using NCBI Batch CD-Search programs (E value < 1e^-2^, https://www.ncbi.nlm.nih.gov/Structure/bwrpsb/bwrpsb.cgi, accessed on 10 March 2023) and SMART (https://smart.embl.de/, accessed on 11 March 2023) software (Letunic et al., 2021). Those protein sequences lacking the C2H2-ZFP domain were discarded.

### 2.2 Sequence characterization analysis and chromosomal location

The ExPASy program was used to evaluate the physical and chemical properties of IbZFPs, such as the length of amino acid residues, molecular weight (kDa), isoelectric point (pI), instability index, and aliphatic index of each IbZFP (https://www.expasy.org/, accessed on 14 March 2023) (Artimo et al., 2012). The phosphorylation sites of IbZFPs were detected using NetPhos-3.1 (https://services.healthtech.dtu.dk/services/NetPhos-3.1/, accessed on 15 March 2023) (Blom et al., 1999). The WoLF PSORT website predicted IbZFPs’ subcellular localizations (https://wolfpsort.hgc.jp/aboutWoLF_PSORT.html.en, accessed on 21 March 2023) (Horton et al., 2007). Retrieve the location information of the *IbZFP* genes from the annotated GFF3 file of the Sweetpotato genome and rename it to IbZFP1-IbZFP178 based on their position on the chromosome.

### 2.3 Phylogenetic analysis of IbZFPs

Using the amino acid sequences of 178 IbZFPs in sweetpotato and 176 AtZFPs proteins in *Arabidopsis thaliana*, a phylogenetic tree was constructed using the maximum likelihood (ML) method by MEGA11 with the following parameters: 1000 bootstrap method, Jones-Taylor-Thornton (JTT) model, pairwise deletion (Tamura et al., 2021). The constructed phylogenetic tree was then classified, annotated, and modified by the Chiplot online tool (https://www.chiplot.online/, accessed on 11 May 2023) (Xie et al., 2023).

### 2.4 Collinearity relationship analysis of *IbZFP* genes

Using “One Step MCScanX Super Fast” of TBtools software v1.131 to analyze downloaded genome sequence files and genome structure annotation information files to obtain collinearity information between *IbZFP* genes (Chen et al., 2020). Then, use the “Advanced Circos” function of TBtools software to visualize the collinear relationship between members of the *IbZFP* gene family.

In addition, in order to conduct homology analysis between the *IbZFP* genes and genes from other plant species, the genome sequences and annotation files of *Ipomoea triloba* and *Ipomoea trifida* were downloaded from the diploid sweetpotato genome website (http://sweetpotato.uga.edu/), and the genome sequences and annotation files of *Arabidopsis thaliana*, *Oryza sativa*, *Solanum tuberosum*, *Manihot esculenta*, *Zea mays*, and *Hordeum vulgare* were downloaded from the Ensemble Plants website (http://plants.ensembl.org/index.html, accessed on 23 May 2023). The collinearity analysis and visualization of these eight plants with the *IbZFP* genes were completed using TBtools software.

### 2.5 Gene structures and conserved motifs analysis of *IbZFP* genes

Tbtools software (v1.131) was used to analyze the GFF annotation files of sweetpotato genome information and visualize the exon-intron structure. The conserved motifs of IbZFP were analyzed using the default parameters of the online website MEME 5.5.2 (https://meme-suite.org/meme/tools/meme, accessed May 21, 2023).

### 2.6 Cis-acting elements in promoter regions analysis of *IbZFP* genes

Use the PlantCARE online website (http://bioinformatics.psb.ugent.be/webtools/plantcare/html/, accessed on 15 June 2023) to analyze the 2000 bp sequence upstream of the start codon of the *IbZFP* gene (Lescot et al., 2002). After screening and simplification, TBtools software was used for visualization.

### 2.7 Expression patterns analysis of *IbZFP* genes

Transcriptome data on SR expansion of two different varieties of sweetpotato from a previous study with NCBI project ID PRJNA756699, including J25_D1, J25_D2, J25_D3, J29_D1, J29_D2, and J29_D3 (Du et al., 2023). In addition, transcriptome data of salt tolerant and salt sensitive sweetpotato varieties under salt stress were obtained from previous studies under NCBI project number PRJNA552932 (Qin et al., 2020), including NL54_0 h, NL54_0.5 h, NL54_6 h, NL54_12 h, J26_0 h, J26_0.5 h, J26_6 h, and J26_12 h. The Chiplot online tool is used to generate heatmaps.

### 2.8 Analysis of *IbZFP* genes expression in different tissues, under stress, and hormone effects

The seedlings of sweetpotato variety ‘Jishu26’ were collected from the Crop Research Institute, Shandong Academy of Agricultural Sciences, China. Seedlings grow in Hoagland solution under a light cycle of 26°C, 16 h of illumination, and 8 h of darkness. When seedlings have 5 to 6 functional leaves and 8 to 10 centimeters of adventitious roots, they are subjected to four different treatments. Hoagland’s solution containing 150 mmol/L NaCl, 20% PEG 6000, 100 mmol/L ABA and 50 mg/L GA3 was used respectively, treated fibrous roots were collected after 0, 3, 6, 12, 24 and 48 h (Hou et al., 2021a). Fibrous roots treated with liquid nitrogen freezing were used to extract total RNA from the sample using RNA isolator Total RNA Extraction Reagent (Vzayme, Nanjing). cDNA was obtained through reverse transcription using a reverse transcription kit (Takara, Beijing), and used as a template. The CFX Connect real-time system (Bio-RAD) and ChamQ Universal SYBR qPCR Master Mix (Vazyme, Nanjing) were used for qRT-PCR. The reaction procedure is 95°C for 30 seconds, followed by 40 cycles of 95 °C for 10 seconds and 60°C for 15 seconds. Using the *Ibactin* gene as an internal reference, **Supplementary Table S5** lists the primer sequences of the examined genes. The experiment was repeated 3 times, and the data was calculated using the 2^-△△CT^ method (He et al., 2023).

### 2.9 Subcellular localization of IbZFPs

The coding sequences of several *IbZFP* genes were inserted into the pCAMBIA1300-GFP vector using homologous recombination method, and the constructed recombinant plasmid was transformed into *Agrobacterium tumefaciens* EHA105. It was then transiently expressed in tobacco leaf (*Nicotiana benthamiana*) cells through injection. Take the transformed GFP leaves as the control. Determine nuclear localization using DAPI staining, observe and take photos using a Laser confocal microscope (Olympus) 2-3 days after injection (Hou et al., 2021b).

### 2.10 Overexpressing *IbZFP105* in *Arabidopsis thaliana*

The coding sequence of IbZFP105 was inserted into the pCAMBIA1300 vector through homologous recombination, and transformed into *Arabidopsis thaliana* through *Agrobacterium tumefaciens* (EHA105) mediated transformation. Possible transgenic strains were obtained through HYG screening, and identified at the DNA and RNA levels using PCR and RT-PCR. The obtained transgenic lines were screened through planting to obtain T3 generation homozygous lines for subsequent experiments.

## 3 Results

### 3.1 Identifcation and phylogenetic analysis of *IbZFP* gene family

In this study, we identified 178 IbZFPs in the sweetpotato genome database and consistently named IbZFP1-IbZFP178 according to their chromosomal locations (**Supplementary Table S1**). The molecular characteristics of all proteins were analyzed, including the number of amino acid (aa) residues, molecular weight (MW), isoelectric point (pI), subcellular localization and phosphorylation sites, *etc*. (**Supplementary Table S1**). IbZFPs vary in length from 68 (IbZFP122) to 1888 (IbZFP8) amino acid residues, correspondingly, with molecular weights ranging from 7.5 to 211.1 kDa. Theoretical pI ranged from 4.05 (IbZFP98) to 11.54 (IbZFP178), and the isoelectric point of 55 IbZFPs was less than 7, indicating that most of the IbZFPs were basic proteins. There are 11 IbZFPs with an instability index less than 40, most of which are unstable proteins. The aliphatic index and grand average of hydropathicity showed that all proteins except IbZFP111 were hydrophilic proteins. Subcellular localization prediction showed that except for IbZFP17, which was localized in the cytoplasm, and IbZFP114 was localized in the chloroplast, all other IbZFPs were localized in the nucleus. The analysis of IbZFP phosphorylation sites showed that IbZFPs have multiple possible phosphorylation sites, with the least 2 (IbZFP73) and the most 209 (IbZFP8).

**Figure 1:**
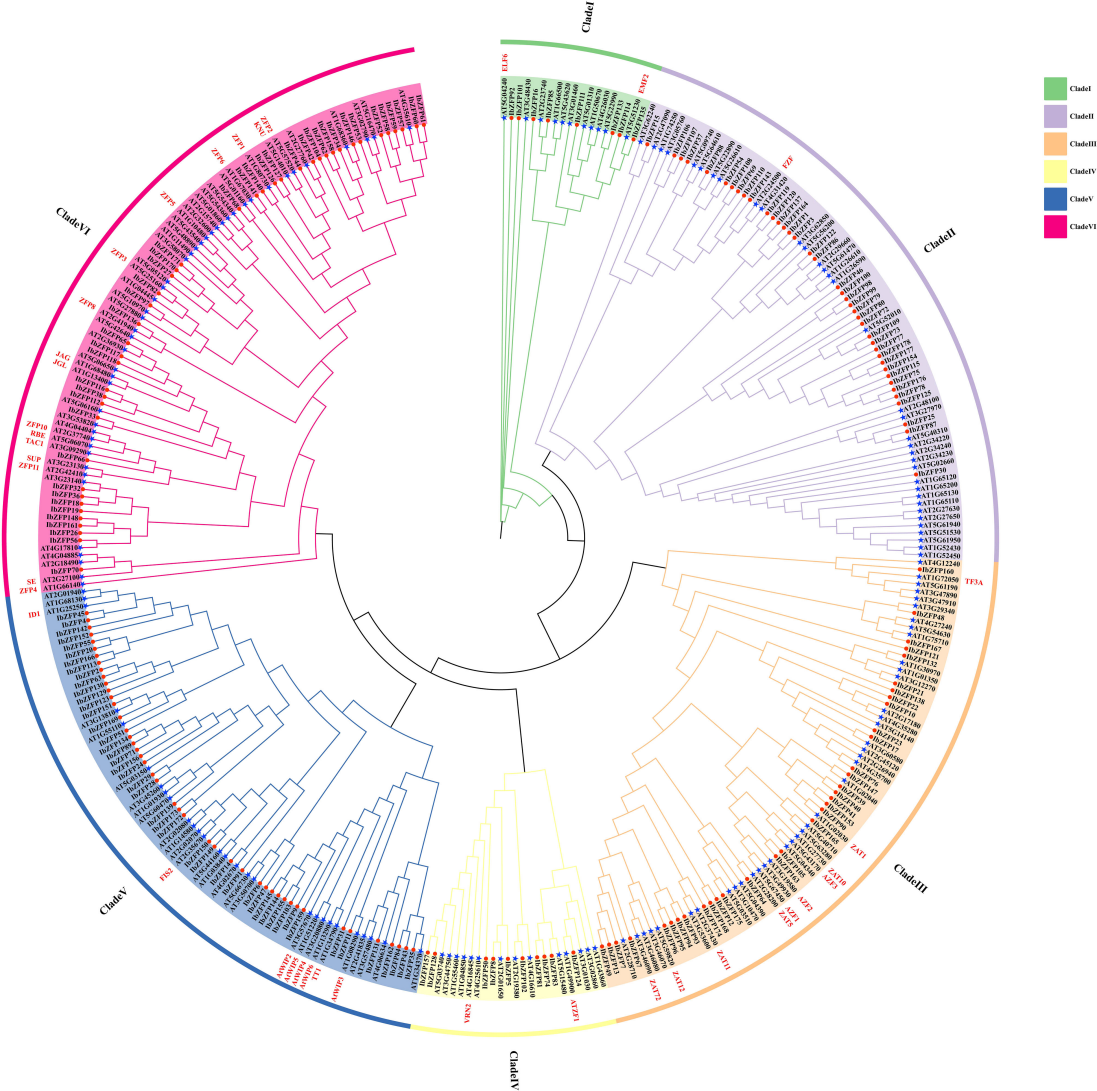
Phylogenetic classification of C2H2 zinc finger proteins (ZFPs) in *Arabidopsis thaliana* and *Ipomoea batatas*. The phylogenetic tree was established using the ML method of 1000 bootstraps. Used different colors to mark different subclasses of the *IbZFP* gene family (branch I-VI) with an arc outside the circular tree. The red circles and blue stars represented ZFPs from *Arabidopsis thaliana* and *Ipomoea batatas*, respectively. *Arabidopsis thaliana* genes that have reported their functions were marked in red font on the outer side of the branch.

In order to explore the phylogenetic relationship of IbZFPs in sweetpotato, the unrooted phylogenetic tree of all 178 IbZFPs and 176 *Arabidopsis thaliana* AtZFPs was constructed by the ML method. Phylogenetic analysis showed that the *IbZFP* gene family could be divided into 6 subfamilies (I-VI) (**Figure 1**), and each clade contained 8, 39, 34, 10, 45, and 42 IbZFPs. Among them, Clade I includes *AtELF6* and *AtEMF2*, which can regulate the flowering of *Arabidopsis thaliana* (Yoshida et al., 2001; Noh et al., 2004). Clade III includes multiple reported functional *Arabidopsis thaliana ZAT* genes, among which *AtZAT10* and *AtZAT12* can enhance salt tolerance in *Arabidopsis thaliana* (Xie et al., 2012), *AtZAT1* can regulate the maturation of the outermost layer cells in the root cap of *Arabidopsis thaliana*, thereby inhibiting its growth (Song et al., 2020), and *AtZAT11* is a dual-function transcriptional regulator that positively regulates primary root growth but negatively regulates Ni^2+^ tolerance (Liu et al., 2014). Clade IV of *VRN2* genes can mediate epigenetic regulation of vernalization in *Arabidopsis thaliana* (Gendall et al., 2001). Clade V includes multiple *Arabidopsis thaliana WIP* members, with *AtWIP1* involved in seed coat development (Sagasser et al., 2002), the *AtWIP2* mutation severely inhibits pollen tube movement, leading to fertility decline in *Arabidopsis thaliana* (Crawford et al., 2007), *AtWIP4* and *AtWIP5* are overexpressed in the pituitary gland and are necessary for the fate of distal stem cells in the root meristem tissue (Crawford et al., 2015). Clade VI includes several *AtZFP* genes with reported functions; for example, *AtZFP1* enhances salt tolerance in *Arabidopsis thaliana* (Han et al., 2014), *AtZFP3* participates in salt and osmotic stress responses (Liu et al., 2020b), and *AtZFP6* plays a key role in regulating trichome (Liu et al., 2020a).

### 3.2 Chromosome distribution and synteny analysis of *IbZFP* genes

**Figure 2:**
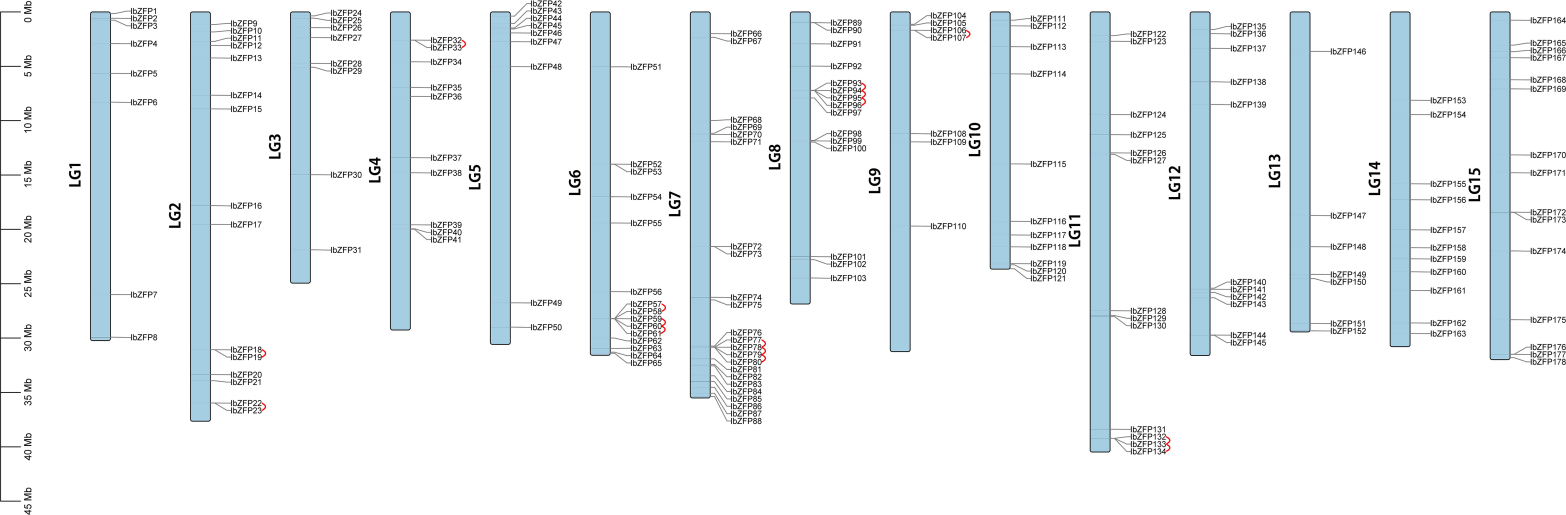
The gene locations of *IbZFP* genes in *Ipomoea batatas*. The scale bar on the left represents the length of the chromosome.

Chromosomal location of *IbZFP* genes showed that 178 members of this gene family were distributed on all 15 chromosomes of *Ipomoea batatas* (**Figure 2**), among which Chr7 had the most with 23 *IbZFP* genes, while Chr9 and Chr13 had the least of 12 *IbZFP* genes.

**Figure 3:**
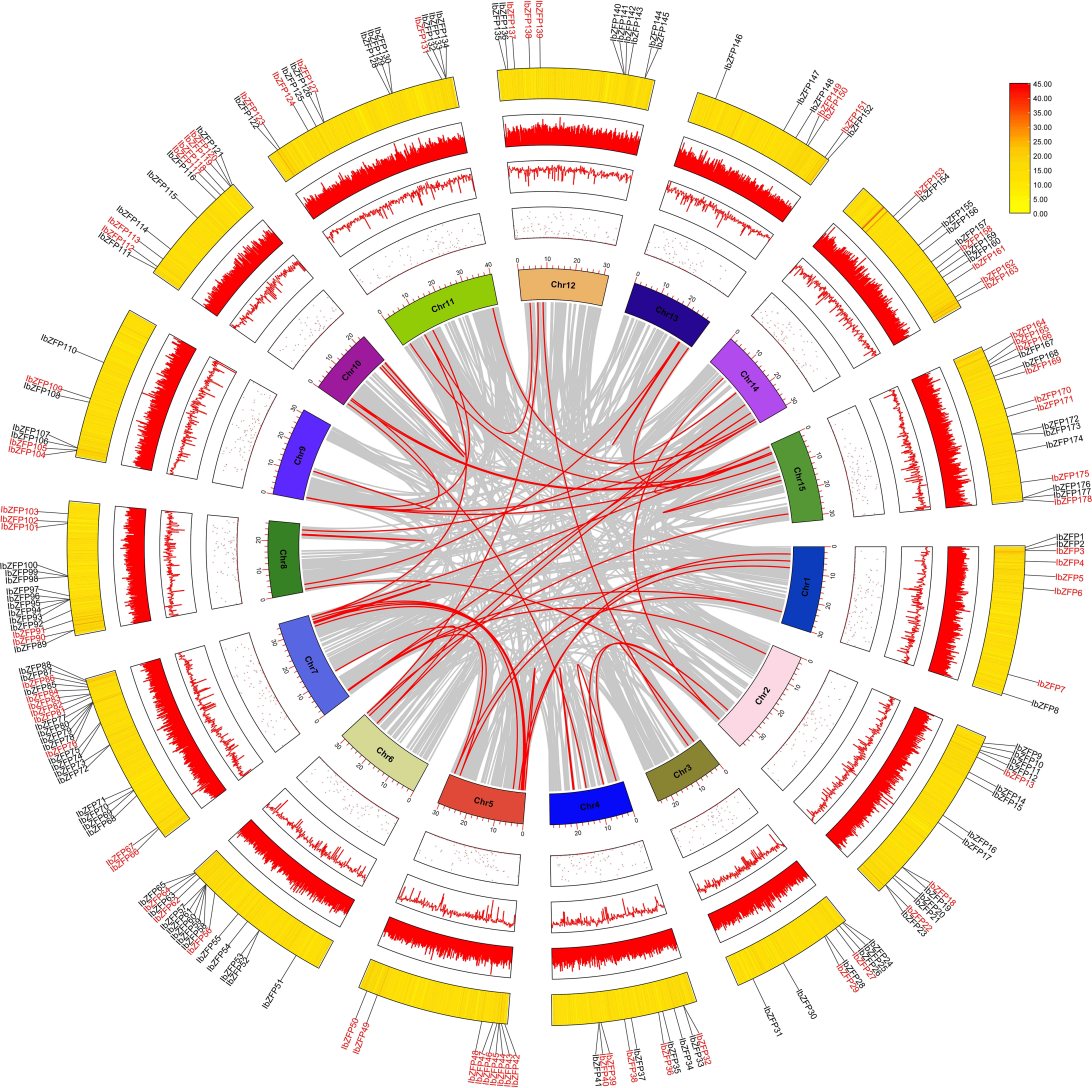
Schematic diagram of the homology relationship of the *IbZFP* genes. Grey represents all collinear fragments in the *Ipomoea batatas* genome, while red represents duplicate *IbZFP* gene pairs. The innermost circle shows the number of chromosomes, while the outer circle points, lines and heat maps show the distribution of unknown bases, the distribution of GC-content and the density of chromosomes, respectively.

Tandem repeat or local replication is the most common mechanism of gene family expansion (Cannon et al., 2004). We identified 15 tandem duplication events containing 24 *IbZFP* genes on Chr2, 4, 6, 7, 8, 9, and 11 (**Figure 2**). Moreover, some *IbZFP* genes participate in more than one tandem repeat event, such as *IbZFP58*, *59*, *60*, *78*, *79*, *etc*. In addition, all genes in tandem repeat events come from the same subfamily, which indicates that the subfamily classification of the evolutionary tree is accurate.Apart from that, collinearity analysis of the *IbZFP* gene family showed that a total of 71 *IbZFP* genes were involved in 46 segment repeat events (**Figure 3**), such as *IbZFP4* on Chr1 and *IbZFP45* on Chr5, *IbZFP13* on Chr2 and *IbZFP67* on Chr7, *IbZFP43* on Chr5, and *IbZFP84* on Chr7. These segmental duplication events are distributed across all 15 chromosomes, with Chr7 containing up to 15 *IbZFP* genes, followed by Chr5 containing 11, and Chr3, Chr9, and Chr12 containing at least 3 *IbZFP* genes, respectively. These results indicate that the evolution of the *IbZFP* gene family is related to gene duplication events, and tandem and segmental repeats play an important role in the amplification of the *IbZFP* genes.

**Figure 4:**
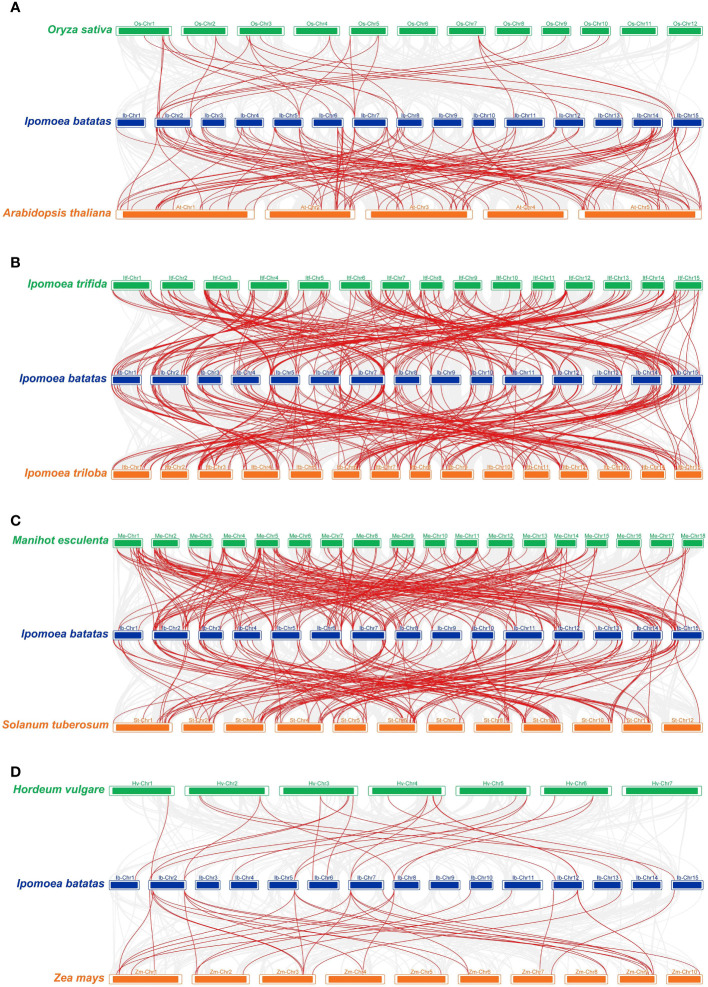
Synteny analyses of *IbZFP* genes between sweetpotato and eight representative plant species. **(A)** *Arabidopsis thaliana* and *Oryza sativa*, **(B)** *Ipomoea trifida* and *Ipomoea triloba*, **(C)** *Solanum tuberosum* and *Manihot esculenta*, **(D)** *Zea mays* and *Hordeum vulgare*. Blue represents the chromosomes of sweetpotato, while green and orange represent the chromosomes of the other two species compared. The red line connecting two different chromosomes highlights the *IbZFP* gene pairs in sweetpotato and other plant genomes.

In order to further infer the origin and evolutionary mechanism of the *IbZFP* genes in sweetpotato, we analyzed and compared the homology of *ZFP* genes between sweetpotato and several different species, including wild varieties *Ipomoea trifida* and *Ipomoea triloba* that are closely related to sweetpotato, two commonly used model plants *Arabidopsis thaliana* and *Oryza sativa*, two edible rhizome plants *Solanum tuberosum* and *Manihot esculenta*, and two main grain plants *Zea mays* and *Hordeum vulgare* (**Figure 4**; **Supplementary Table S2**). Results display that 124 and 122 *IbZFP* genes showed syntenic connections with *Ipomoea trifida* and *Ipomoea triloba*, followed by *Manihot esculenta* (102), *Solanum tuberosum* (98), and *Arabidopsis thaliana* (67). The ones with less collinearity are *Oryza sativa* (18), *Hordeum vulgare* (17), and *Zea mays* (16). It is worth noting that *IbZFP* genes have the strongest collinearity with *Ipomoea trifida* and *Ipomoea triloba*. It may be that *Ipomoea trifida* and *Ipomoea triloba* are closely related wild species of sweetpotato, and *IbZFP* has much higher collinearity with edible rhizome plants such as *Manihot esculenta* and *Solanum tuberosum* than the model plant *Arabidopsis thaliana*, Poaceae plants *Oryza sativa*, *Hordeum vulgare*, and *Zea mays*, which may indicate that the *IbZFP* gene family is relatively conservative in the evolution of tuber or root tuber crops and may play a role in the expansion process of tubers or root tubers.

### 3.3 Gene structure and cis-acting elements analysis of *IbZFP* genes

**Figure 5:**
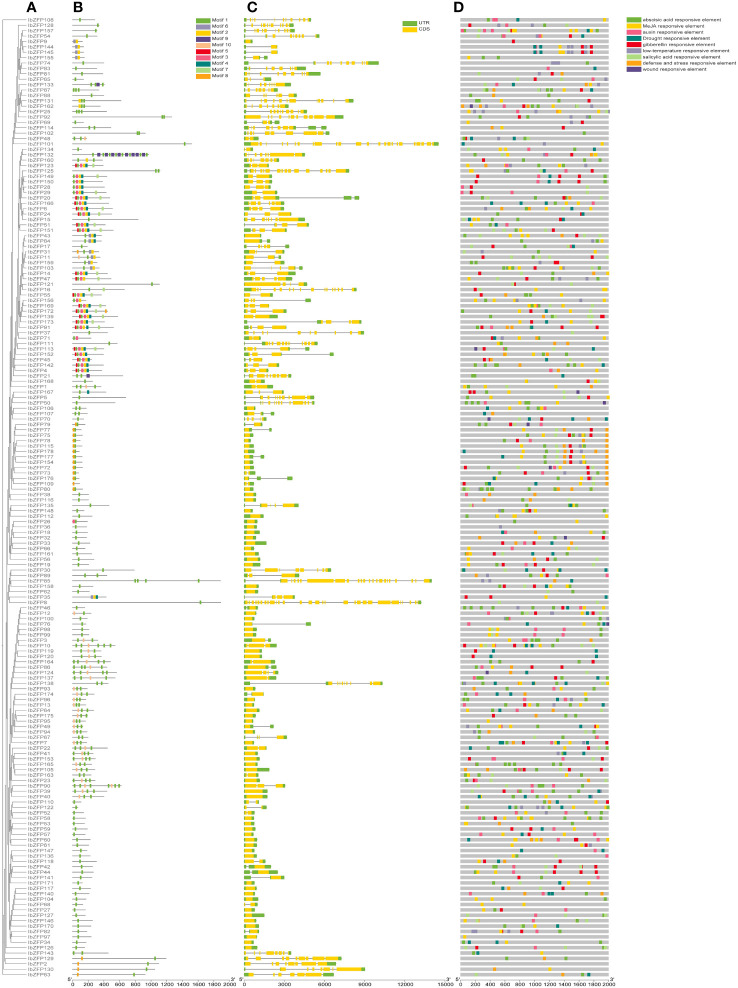
Gene structure, motif composition and promoter cis-elements analysis of *IbZFP* genes. **(A)** The phylogenetic tree of IbZFPs was constructed using the ML method. **(B)** The distribution of ten largely conserved motifs found in IbZFPs is shown in the graph below **(C)** The exon/intron structures of IbZFPs, were predicted by TBtools. **(D)** Predicting cis-elements in the *IbZFP* genes promoter through the PlantCARE website.

In order to understand the structural diversity of these IbZFPs, we generated a phylogenetic tree using all IbZFPs (**Figure 5A**) and compared their exon/intron composition and conservative domain of IbZFPs (**Figure 5C**). The results showed that 65 of the 178 IbZFPs did not contain intron, accounting for 36.5% of the total number of IbZFPs. On the contrary, there are 11 IbZFPs with more than 10 intron, of which there are 28 intron in IbZFP8. The number of intron in the remaining 102 IbZFPs ranges from 1 to 10. The exon/intron structure of the sequence reflects the structural diversity and complexity of IbZFPs. In addition, in the study of the conserved domains of the IbZFPs sequence, 10 conserved motifs were obtained (**Figure 5B**), among which motifs 1, 2, and 3 conform to the sequence characteristics of the C2H2 zinc finger (**Supplementary Figure S1**). Except for IbZFP9, 144, 145, and 155, which only have motif 2, motif 1 exists in all IbZFPs. We found the plant specific Q-type C2H2-ZFPs symbol “QALGGH” in motif 1, indicating that motif 1 should be a conserved and important motif in the *IbZFP* gene family of sweetpotato (Cui et al., 2022).

To further understand the potential regulatory mechanisms of the *IbZFP* genes in response to stress and various hormones. The 2000 bp promoter region upstream of the *IbZFP* genes was analyzed using PlantCARE online website, and nine cis-regulatory elements related to stress or hormone were identified (**Figure 5D**; **Supplementary Table S3**). Among them, 137 promoters of the *IbZFP* genes (77%) had elements corresponding to stress, such as low-temperature responsive elements, drought responsive elements, wound responsive elements, and defense and stress responsive elements. 174 promoters of the *IbZFP* genes (97.7%) had hormone responsive elements (auxin, gibberellin, abscisic acid, MeJA, and salicylic acid responsive elements). More cis-elements were found in the promoters of *IbZFP80* (21), *IbZFP10* (19), and *IbZFP157* (19), while no cis-elements related to stress and hormone response were found in the promoter of *IbZFP2*. The analysis of the promoter of *IbZFP* genes showed that these cis-elements may have a potential role in affecting the development and responding to abiotic stress of sweetpotato.

### 3.4 Expression patterns analysis of *IbZFP* genes during SR development and salt stress

In order to study the expression patterns of *IbZFP* genes during SR expansion of sweetpotato, we used the transcriptome data of two different sweetpotato varieties at the early stage of SR expansion to analyze their expression levels (Du et al., 2023).

**Figure 6:**
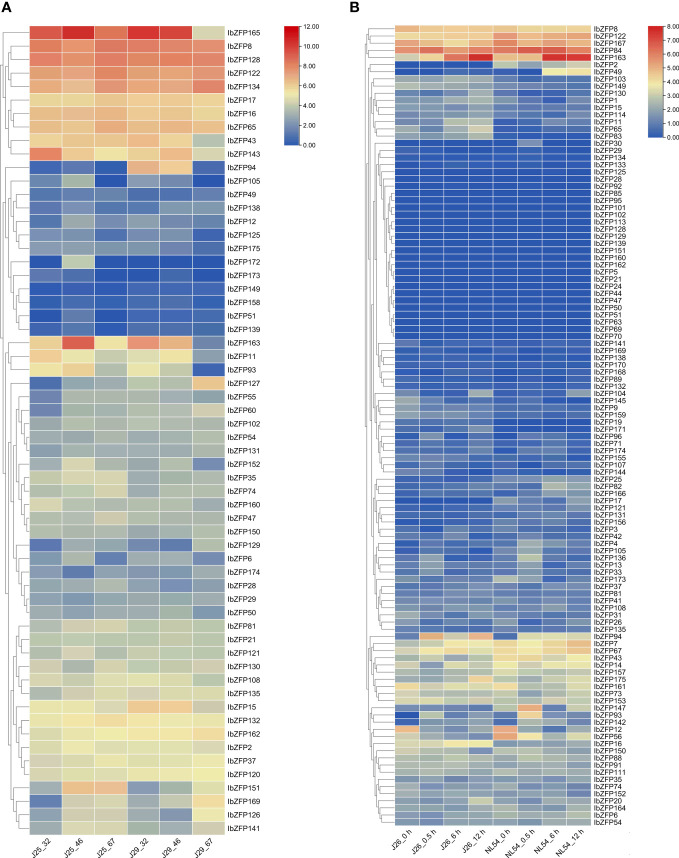
**(A)** Expression profiles of *IbZFP* genes during the development of sweetpotato storage roots. The horizontal axis represents the samples of sweetpotato varieties ‘Jishu25’ and ‘Jishu29’ at 32, 46, and 67 DAP, respectively **(B)** Expression profiles of *IbZFP* genes in sweetpotato under salt stress. The abscissa represents the samples of sweetpotato varieties ‘Jishu26’ and ‘NL54’ under salt treatment at 0, 0.5, 6, and 12 hours. All ratios undergo a log_2_ transformation, with red blocks indicating high relative expression levels and blue blocks indicating low relative expression levels.

We identified 44 *IbZFP* genes from the SR transcriptome data 32 days, 46 days, and 67 days after planting (DAP) of two sweetpotato varieties (‘Jishu25’ and ‘Jishu29’), and displayed the expression levels of these genes with a heatmap according to FPKM (Fragments Per Kilobase of exon model per Million mapped fragments) values (**Figure 6A**). The research results showed that some *IbZFP* genes, such as *IbZFP37*, *49*, *74*, *135*,*151*, and *169*, gradually increase in expression during SR development in two different varieties, while some genes (*IbZFP15* and *IbZFP160*) gradually decrease in expression levels during SR development in different varieties. In addition, we noticed that *IbZFP8* and *IbZFP128* exhibited significantly higher transcriptional accumulation than other *IbZFP* genes during SR development in different varieties (**Supplementary Table S4**). These results indicate that the *IbZFP* genes exhibit different expression patterns during SR development in sweetpotato, and some *IbZFP* genes may play an important regulatory role in the early expansion process of sweetpotato SR.

Similarly, in order to study the possible function of *IbZFP* genes in sweetpotato responses to stress, the transcriptome data of salt tolerant and salt sensitive sweetpotato varieties (‘Jishu26’ and ‘NL54’) under salt stress were used to analyze the expression pattern (Qin et al., 2020) of *IbZFP* genes under salt stress.

We identified 113 *IbZFP* genes in the sample transcriptome data of salt tolerant and salt sensitive varieties 0 h, 0.5 h, 6 h and 12 h after salt treatment, and mapped the expression calorimetry according to the FPKM value (**Figure 6B**). The research results showed that among two different salt stress resistant sweetpotato varieties, 21 *IbZFP* genes (18.6%) were not detected for expression in any treatment (FPKM value equals 0) (**Supplementary Table S4**), 8 *IbZFP* genes (such as *IbZFP9*, *41*, *63*, and *142*) were significantly upregulated after salt stress, and 14 *IbZFP* genes (such as *IbZFP12*, *56*, and *161*) were significantly downregulated after salt stress. It is worth noting that we found that 23 *IbZFP* genes (such as *IbZFP11*, *16*, and *65*) were expressed at higher levels in the salt tolerant variety ‘Jishu26’ compared to the salt sensitive variety ‘NL54’, while 19 *IbZFP* genes (*IbZFP2*, *14*, *43*, etc.) were expressed at higher levels in salt sensitive varieties. It is speculated that these genes may be related to the regulation of salt tolerance in sweetpotato.

### 3.5 Expression analysis of *IbZFP* genes in different tissues, hormones, and stress conditions and subcellular localization

**Figure 7:**
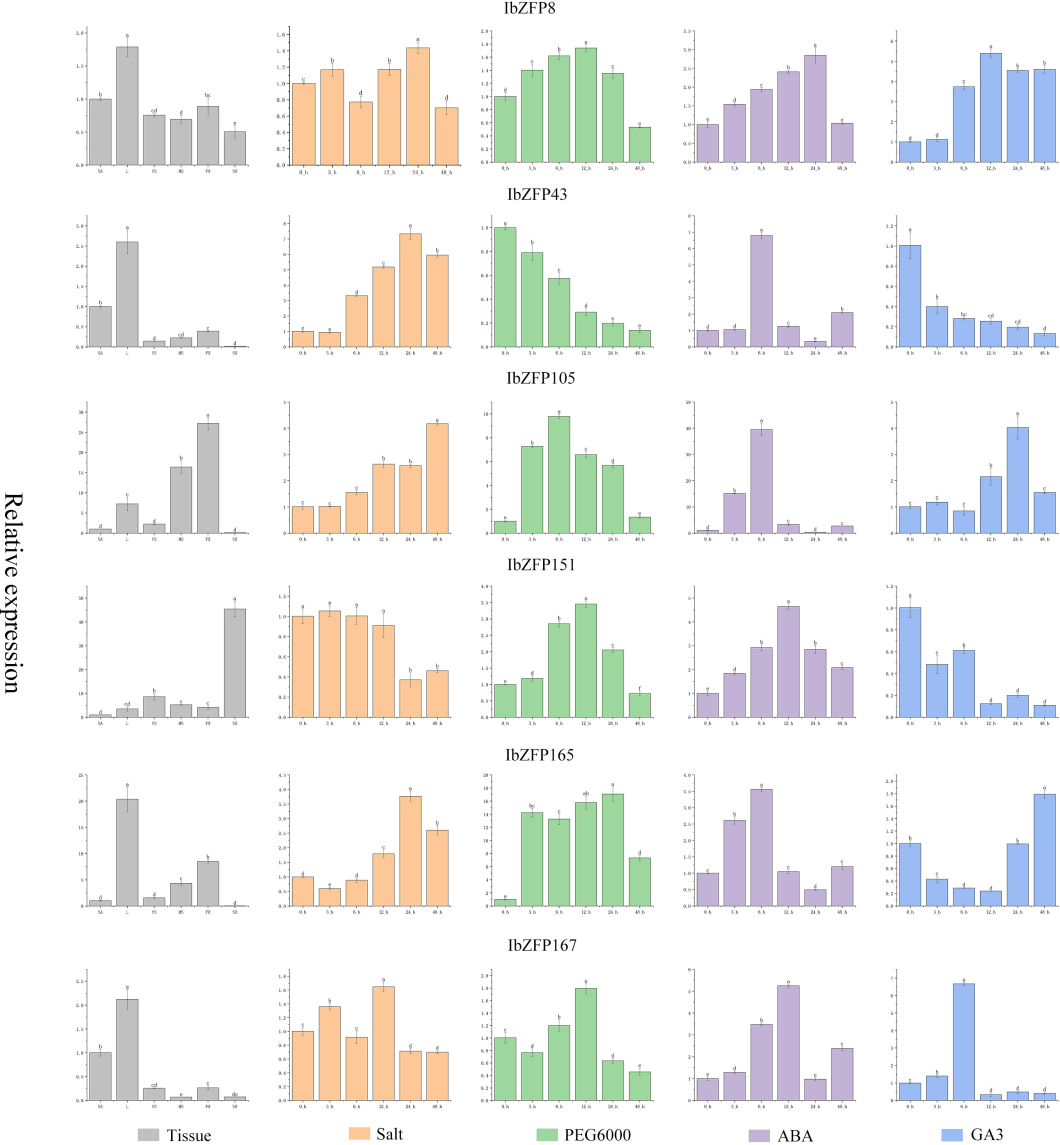
Expression profile of *IbZFP* genes in different tissues, under stress and hormone effects. The gray part in the figure represents the expression profile of *IbZFP* genes in different tissues of sweetpotato (where SA represents stem apex, L represents leaves, YS and MS represent young and mature stem, FR and SR represent fibrous root and storage root, respectively). And the orange part of the figure shows the expression profile of *IbZFP* genes under NaCl induced salt stress, the green part represents the expression profile under PEG6000 induced drought stress, the purple part represents the expression profile under ABA hormone induction, and the blue part represents the expression profile under GA_3_ induction.

Based on the results of the previous analysis, we screened 6 *IbZFP* genes (*IbZFP8*, *43*, *105*, *151*, *165*, and *167*) and examined their expression patterns in various tissues under abiotic stress (PEG6000-induced drought stress and NaCl-induced salt stress) and hormone-induced stress (ABA and GA_3_) using qRT-PCR (**Figure 7**). This allowed us to better understand the role of the *IbZFP* gene in the development of the sweetpotato SR and stress tolerance.

The research results show that the *IbZFP* genes exhibits different expression patterns in different tissues of sweetpotato, with *IbZFP8*, *43*, *165*, and *167* having the highest expression levels in leaves, while *IbZFP105* and *IbZFP151* have the highest expression levels in FR and SR. This result suggests that the *IbZFP* genes may play a role in the development of sweetpotato. And under NaCl induced salt stress, except for the downregulation of *IbZFP151* expression with NaCl treatment time, the expression levels of *IbZFP43*, *105*, and *165* showed significant upregulation with stress time. Under drought stress, the expression of *IbZFP* genes showed a trend of upregulation and then downregulation, except for *IbZFP43*. Under ABA induction, the *IbZFP* genes showed a similar trend of first increasing and then decreasing. Different from ABA induction, the expression of the *IbZFP* genes showed a completely different trend under GA_3_ induction. The expression of *IbZFP43* and *151* decreased significantly under GA_3_ induction, while the expression of *IbZFP165* decreased first and then increased with GA_3_ treatment time.

**Figure 8:**
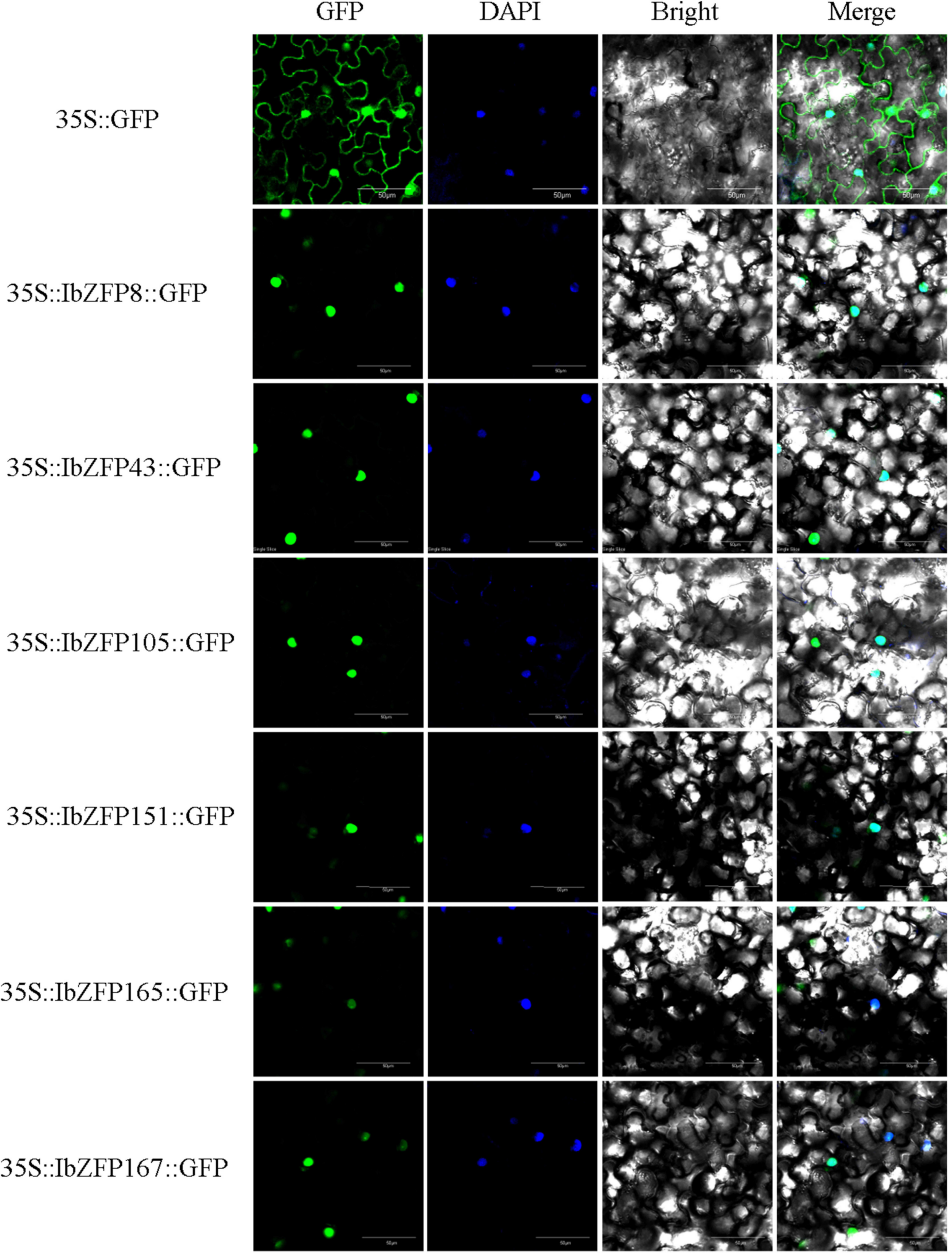
Subcellular localization of IbZFPs. Inject tobacco (*Nictiana benthamiana*) leaves after transforming *Agrobacterium tumefaciens* using recombinant plasmid (IbZFP-GFP) and empty control (GFP). The figure shows confocal images of GFP fluorescence, nuclear localization (DAPI), bright field, and composite field.

We previously predicted the subcellular localization information of all IbZFPs through the website (**Supplementary Table S1**), and it is important to determine whether transcription factors are located in the cell nucleus and study their regulatory function. Therefore, we recombined 6 IbZFPs (IbZFP8, 43, 105, 151, 165, and 167) onto GFP vectors to verify their subcellular localization results (**Figure 8**). The results showed that all six IbZFPs were located in the nucleus.

### 3.6 Overexpression of *IbZFP105* in *Arabidopsis thaliana*

**Figure 9:**
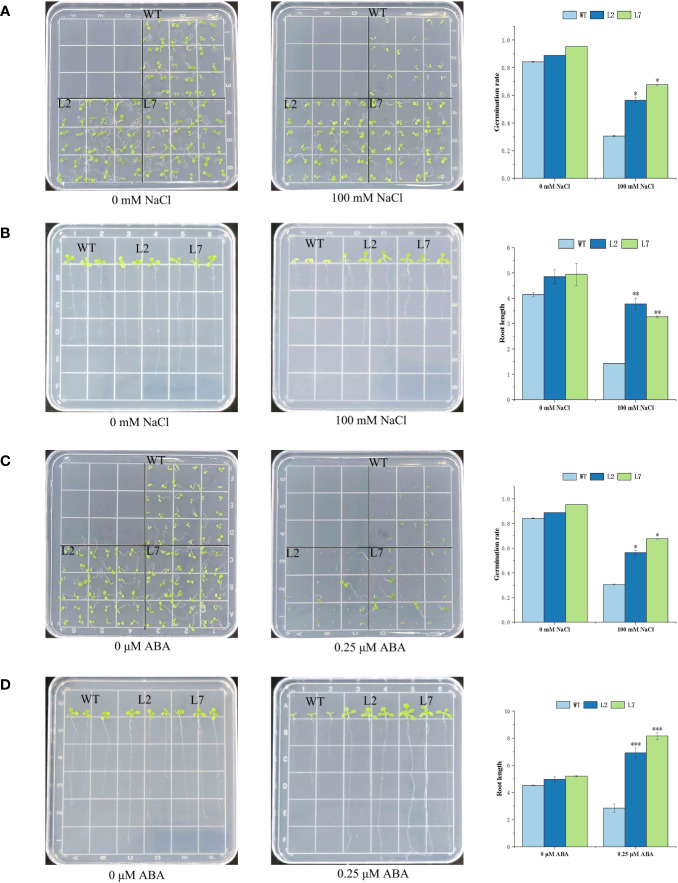
Functional Analysis of Overexpressed *IbZFP105* Gene in *Arabidopsis thaliana*. **(A, B)** Salt tolerance identification of germination and growth status of OE-L2, OE-L7, and WT in 1/2 MS medium containing 100 mmol/L NaCl. **(C, D)** OE-L2, OE-L7, and WT were subjected to ABA stress identification in 1/2 MS medium containing 0.25 µmol/L of ABA. The data consists of three independent biological replicates (* represents p<0.05, * * represents P<0.01, and * * * represents P<0.001).

To verify the function of the *IbZFP* genes in stress resistance, we cloned the *IbZFP105* gene in ‘Jishu26’ and constructed an overexpression vector pCAMBIA1300-*IbZFP105*, which was transferred into *Arabidopsis thaliana* (Col-0) through the *Agrobacterium* mediated inflorescence soaking method. Verify the positive lines by PCR amplification of the target gene and RT-PCR detection of gene expression levels (**Supplementary Figure S1**). We selected L2 and L7 with the highest relative expression levels from the 8 positive lines obtained for subsequent functional validation. To verify whether *IbZFP105* responds to salt stress, we planted wild-type (WT) and transgenic *Arabidopsis thaliana* in a 1/2 MS medium containing 100 mmol/L NaCl (**Figures 9A, B**) and 0.25 μmol/L of ABA (**Figures 9C, D**), measure its germination rate and root length after 15 DAP. The results showed that at 15 DAP, the OE lines of *IbZFP105* showed significantly better germination rate and growth status than the WT under salt stress and ABA treatment. This indicates that the heterologous expression of *IbZFP105* enhances salt tolerance and ABA tolerance in *Arabidopsis thaliana*, suggesting that *IbZFP105* enhances plant salt tolerance by responding to the ABA pathway.

## 4 Discussion

C2H2-ZFPs are one of the most important transcription factor families in higher plants, playing a crucial role in plant development and stress resistance (Han et al., 2021; Liu et al., 2022a; Liu et al., 2022b). Previous reports have shown that they can play important roles in *Arabidopsis thaliana*, wheat, grapevine, sorghum, and cotton (Englbrecht et al., 2004; Salih et al., 2019; Arrey-Salas et al., 2021; Cui et al., 2022; Wu et al., 2022). However, there are few reports on the C2H2-ZFPs in sweetpotato. In this study, we conducted a genome-wide study on the C2H2-ZFP family using genomic data from hexaploid sweetpotato Taizhong6. A total of 178 IbZFPs were screened and identified, encoding proteins with at least one C2H2-ZFP conserved motif.

By constructing phylogenetic evolution trees of *Arabidopsis thaliana* and sweetpotato ZFPs, we divided IbZFPs into six clades and found multiple reported functional *Arabidopsis thaliana* ZAT proteins, such as ZAT1, 5, and 10 (Xie et al., 2012; Liu et al., 2014; Yin et al., 2017), in clade III, which mostly regulate stress resistance in *Arabidopsis thaliana*. We found that IbZFP43, 105, and 165 are closely related to these *Arabidopsis thaliana* ZAT proteins, indicating that these genes may also be involved in the response to stress in sweetpotato.

Then we analyzed the chromosomal localization and gene duplication events of IbZFPs. Gene duplication events, including tandem and segmental duplication, play an important role in the evolution and expansion of gene families (Vision et al., 2000). We investigated the mechanism of member amplification of *IbZFP* genes in sweetpotato and identified 15 tandem repeat events composed of 24 *IbZFP* genes, with some genes participating in multiple tandem repeats. We also identified 46 fragment replicates involving 71 *IbZFP* genes, indicating that segmental gene replicates play important roles in the evolution and diversification of the C2H2 zinc finger gene family in the sweetpotato genome. This result is similar to the analysis of the C2H2 zinc finger gene family in other species, such as poplar and sorghum (Liu et al., 2015; Cui et al., 2022).

By analyzing the collinearity of sweetpotato and other species in the C2H2 zinc finger gene family, we have discovered an interesting phenomenon where the *IbZFP* genes have 124 putative orthologous homologues in *Ipomoea trifida*, which is much higher than other plants, possibly due to the closer phylogenetic relationships between sweetpotato and *Ipomoea trifida*. In addition, the *IbZFP* genes have 102 and 98 putative orthologous compounds in cassava and potato, much higher than the model plant *Arabidopsis thaliana*. We believe that this means that the C2H2 zinc finger gene family is relatively conserved in the evolution of tuber or root crops and may play an important regulatory role in the expansion process of tubers or roots.

In addition, the gene structure and conserved motifs of IbZFPs were analyzed, with motifs 1, 2, and 3 being the characteristics of C2H2 zinc fingers. Among them, motif 1 has a conserved QLAGGH sequence, which is the symbol of plant specific Q type C2H2-ZFP. Q type C2H2-ZFP is unique to plants and participates in the growth, development, organogenesis, and response to stress and defense in various plants (Cui et al., 2022), 174 IbZFPs (97.8%) have motif 1, indicating that motif 1 plays a crucial regulatory role in IbZFPs. In addition to C2H2 type motifs, IbZFPs also contain many other motifs, indicating that IbZFPs play a wide range of roles in sweetpotato.

We analyzed the expression profile of the *IbZFP* genes in the early development and salt stress transcriptome of sweetpotato SRs, identified several *IbZFP* genes that may be related to early development and salt stress tolerance of sweetpotato SRs. Subsequently, we cloned these genes in sweetpotato and analyzed their tissue specificity, expression patterns under drought and salt stress, and expression patterns under plant hormone ABA and GA_3_ treatment, then their subcellular localization was verified in tobacco leaf cells. We found that the *IbZFP* genes exhibits different expression patterns in different parts of sweetpotato. Some genes are highly expressed in the leaves, while others are highly expressed in FR or SR. Multiple species such as cotton and wheat have also reported this phenomenon, indicating that the C2H2-ZFP may play a crucial role in the formation and development of various plant tissues.

Moreover, through RT-PCR analysis of the expression patterns of several *IbZFP* genes under abiotic stress and hormone treatment, we found that several *IbZFP* genes exhibited different expression patterns induced by different stresses and hormones. We selected the *IbZFP105* gene with the most significant difference in expression levels under salt stress and ABA treatment, constructed its overexpression vector, and transformed it into *Arabidopsis thaliana*, through experiments on the germination rate and growth status of *Arabidopsis thaliana* seeds under salt and ABA stress, we found that the OE lines exhibited stronger salt tolerance and ABA stress resistance. Therefore, we speculate that IbZFP105 can improve plant salt tolerance by responding to ABA signals.

## Data availability statement

The original contributions presented in the study are included in the article/**Supplementary Material**, further inquiries can be directed to the corresponding authors.

## Author contributions

TD: Conceptualization, Data curation, Validation, Visualization, Writing – original draft. YZ: Validation, Writing – review & editing. ZQ: Project administration, Software, Writing – review & editing. AL: Formal Analysis, Funding acquisition, Writing – review & editing. QW: Formal Analysis, Funding acquisition, Writing – review & editing. ZL: Investigation, Writing – review & editing. FH: Conceptualization, Resources, Supervision, Writing – original draft, Writing – review & editing. LZ: Methodology, Resources, Writing – review & editing.
