# Supplementary material for: Genome-wide identification of the C2H2 zinc finger gene family and expression analysis under salt stress in sweetpotato
Source: Front Plant Sci. 2023 Dec 13;14:1301848. doi: 10.3389/fpls.2023.1301848 (PMC10752007; doi:10.3389/fpls.2023.1301848)

**Supplementary Figure S1**. Identification of overexpressed *IbZFP105* Gene in *Arabidopsis thaliana.* (A) PCR identification results of *IbZFP105* gene in OE lines. The *IbZFP105* gene can be amplified in the OE lines but cannot be amplified in the WT line. (B) RT-PCR identification of the expression level of the *IbZFP105* gene in the OE lines.


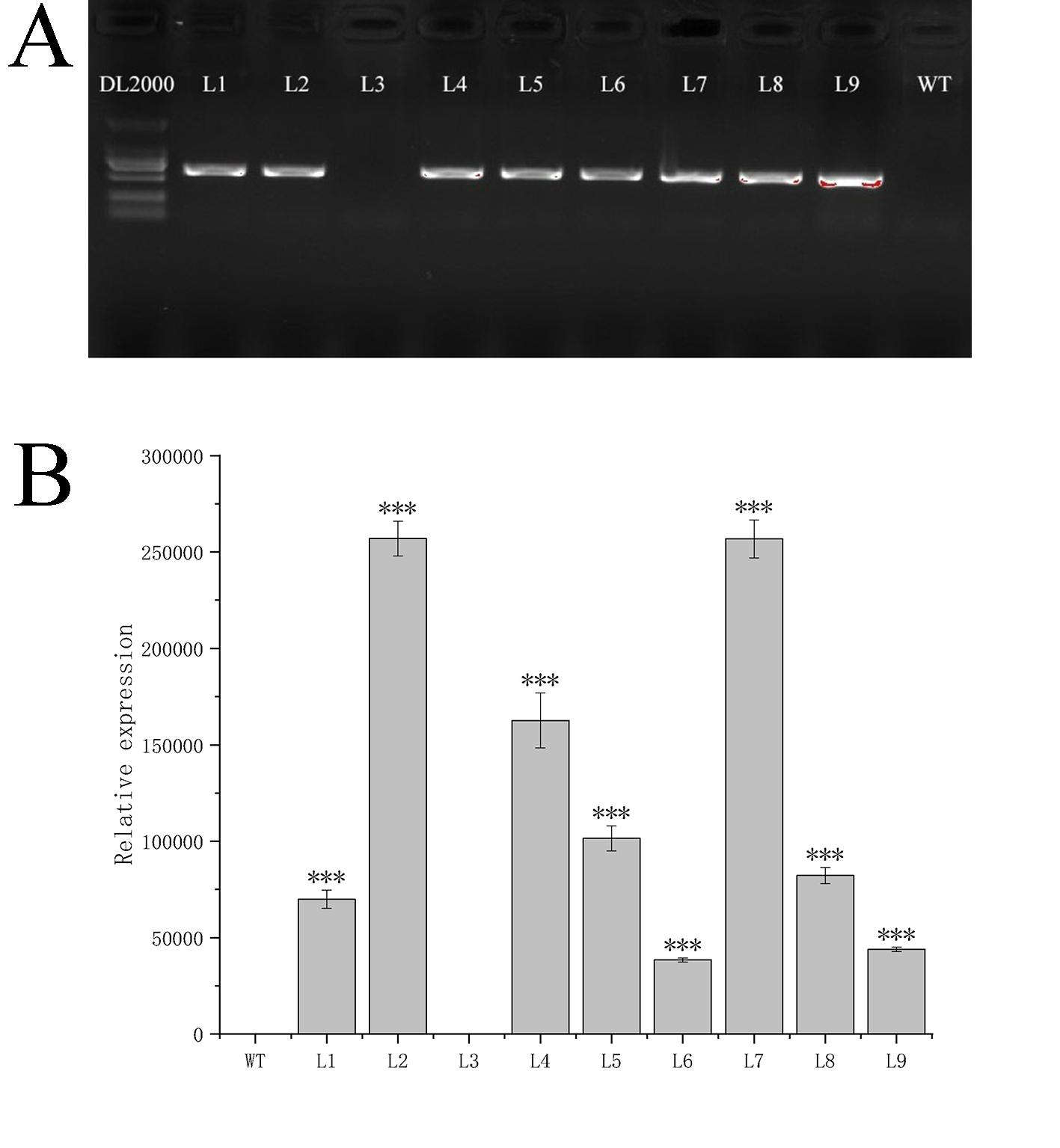

Supplement: Supplementary file 1 [file DataSheet_1.zip › Datasheet 1.docx]
